# Research progress of extracellular vesicles as biomarkers in immunotherapy for non-small cell lung cancer

**DOI:** 10.3389/fimmu.2023.1114041

**Published:** 2023-04-21

**Authors:** Yang Ge, Ting Ye, Siyun Fu, Xiaoying Jiang, Hang Song, Bin Liu, Guoquan Wang, Jinghui Wang

**Affiliations:** ^1^ Graduate School, Anhui University of Chinese Medicine, Hefei, China; ^2^ Department of Cellular and Molecular Biology, Beijing Chest Hospital, Capital Medical University/Beijing Tuberculosis and Thoracic Tumor Research Institute, Beijing, China; ^3^ Department of Science and Technology, Beijing Chest Hospital, Capital Medical University, Beijing Tuberculosis and Thoracic Tumor Research Institute, Beijing, China; ^4^ School of Integrated Chinese and Western Medicine, Anhui University of Chinese Medicine, Hefei, China

**Keywords:** EV, biomarker, immunotherapy, chemoimmunotherapy, TME, NSCLC

## Abstract

Lung cancer is one of the most severe forms of malignancy and a leading cause of cancer-related death worldwide, of which non-small cell lung cancer (NSCLC) is the most primary type observed in the clinic. NSCLC is mainly treated with surgery, radiotherapy, and chemotherapy. Additionally, targeted therapy and immunotherapy have also shown promising results. Several immunotherapies, including immune checkpoint inhibitors, have been developed for clinical use and have benefited patients with NSCLC. However, immunotherapy faces several challenges like poor response and unknown effective population. It is essential to identify novel predictive markers to further advance precision immunotherapy for NSCLC. Extracellular vesicles (EVs) present an important research direction. In this review, we focus on the role of EVs as a biomarker in NSCLC immunotherapy considering various perspectives, including the definition and properties of EVs, their role as biomarkers in current NSCLC immunotherapy, and different EV components as biomarkers in NSCLC immunotherapy research. We describe the cross-talk between the role of EVs as biomarkers and novel technical approaches or research concepts in NSCLC immunotherapy, such as neoadjuvants, multi-omics analysis, and the tumour microenvironment. This review will provide a reference for future research to improve the benefits of immunotherapy for patients with NSCLC.

## 1 Introduction

Lung cancer is the most common type of cancer and a leading cause of cancer-related death in China (1). Lung cancer is classified into two subtypes: small cell lung cancer and non-small cell lung cancer (NSCLC), of which NSCLC accounts for 85% of all lung malignancies (2). Squamous cell carcinoma, adenocarcinoma (AD), and large cell carcinoma are the three forms of NSCLC. AD accounts for around 40% of all lung cancers, squamous cell carcinoma for 25%–30%, and large cell carcinoma for 5%–10% (3). The aetiology of lung cancer remains unclear. Smoking and air pollution are two significant risk factors. Other risk factors, such as occupational exposure (e.g. asbestos), also play a significant role in the development of lung cancer (4). Surgery, radiation, chemotherapy, targeted therapy, and immunotherapy are used for treating NSCLC (2, 5–8). Treatment options vary according to the type and stage of cancer. A majority of patients with NSCLC are diagnosed at stage IV. Surgery is the primary treatment for stage I–IIIA NSCLC, but patients with stage IIIB–IV are generally treated with radiation or chemotherapy because of the metastasis of the tumour. The 5-year overall survival rate for NSCLC is dismal, with 68% for patients in stage IB and 0%–10% for those in stage IVA–IVB (3, 9, 10). Immunotherapy is a novel treatment that has shown promising outcomes and improved patient prognosis. The ligand-receptor interaction is necessary for self-tolerance and physiological immune regulation. Immune checkpoint inhibitors (ICIs) are a novel class of immunotherapy-based drugs that are one of the most regularly employed techniques in tumour immunotherapy for enhancing survival in NSCLC (11). Antibodies of programmed death-1 (PD-1) and its ligand (PD-L1), for example, are utilized for the therapy of NSCLC to stimulate anti-tumour immune responses by preventing inhibitory immunological signals. Conversely, combination chemo-immunotherapy only shows a substantial therapeutic response in patients with NSCLC having more than 50% expression of the PD-L1 biomarker (12). Furthermore, ICI treatment has certain severe side effects and can result in immune-mediated checkpoint inhibitor pneumonia that affects 3%–5% of patients with NSCLC treated with ICIs (13).

Chemo-immunotherapy is now the mainstay for NSCLC treatment, with results often outperforming immunization alone. PD-L1 therapy paired with cytotoxic chemotherapy is often utilized in the treatment of patients with NSCLC. The KEYNOTE-189 and KEYNOTE-407 studies on non-squamous and squamous cancers, respectively, both found that immunotherapy in conjunction with chemotherapy improved progression-free survival as compared to that of chemotherapy alone. In all studies, the control group crossover rate was approximately 50%, indicating that early administration of immunotherapy can deliver significant advantages (12).

CTLA-4, an inhibitory receptor, is located on effector and regulatory T cells that competes with CD28 for binding to control the immune response. Tumour cells can accomplish immunological escape by activating CTLA-4, which inactivates T lymphocytes according to previous research. The anti-CTLA-4 ICI, ipilimumab, has been proven for the treatment of metastatic melanoma. In advanced clinical studies of NSCLC, monoclonal antibodies of ipilimumab have exhibited significant increases in patients’ overall survival. PD-1 and CTLA-4 are complementary co-suppressor receptors that suppress T cell immunological responses. The CheckMate-227 phase III study found that PD-1 with CTLA-4 blockade increased patients’ overall survival (14). Unfortunately, antibodies that neutralize inhibitory factors and cytokines frequently cause toxic side effects, such as diarrhoea, thyroid dysfunction, and hyperglycaemia, as a result of immunotherapy (15). Anti-PD-1 expression in tumour tissue can be employed as a prognostic biomarker in immunohistochemistry studies. However, because of the tumour heterogeneity, possibility for gene expression at many places, and metastasis in various areas, many patients with cancer may not have enough tumour tissue for testing. Moreover, tissue biopsies may not be entirely diagnostic of the tumour phenotype.

Consequently, it is crucial to investigate biomarkers with more universal therapeutic capabilities for advanced therapies of cancer (16).

Immunotherapy has significant limitations, including the lack of particular indicators and small patient group to benefit from it. New biomarker-based, non-invasive, accurate, and safer diagnostic methods are required for the immunotherapy of NSCLC. Current research is concentrated on extracellular vesicles (EVs) with diameters less than 150 nm, known as exosomes. In this review, we will mainly discuss exosomes as one of the EV subtypes.

EVs are intercellular communication vehicles that convey and control the physiological status of cells and are directly involved in the genesis and progression of many diseases (17). They include microvesicles (MVs), apoptotic bodies (ApoBDs), and exosomes (14). Exosomes have been discovered to affect tumour mechanisms. As a biomarker they play an important role in the diagnosis, treatment, and prognosis of NSCLC by modulating the production of miRNAs, lncRNAs, circRNAs, and proteins (18–21). In this review, we discuss, the definitions, properties, contents, and separation methods of EVs along with future research directions and challenges.

## 2 Extracellular vesicles: definition, properties, and isolation methods

### 2.1 Definitions and categorization of EV

**Figure 1:**
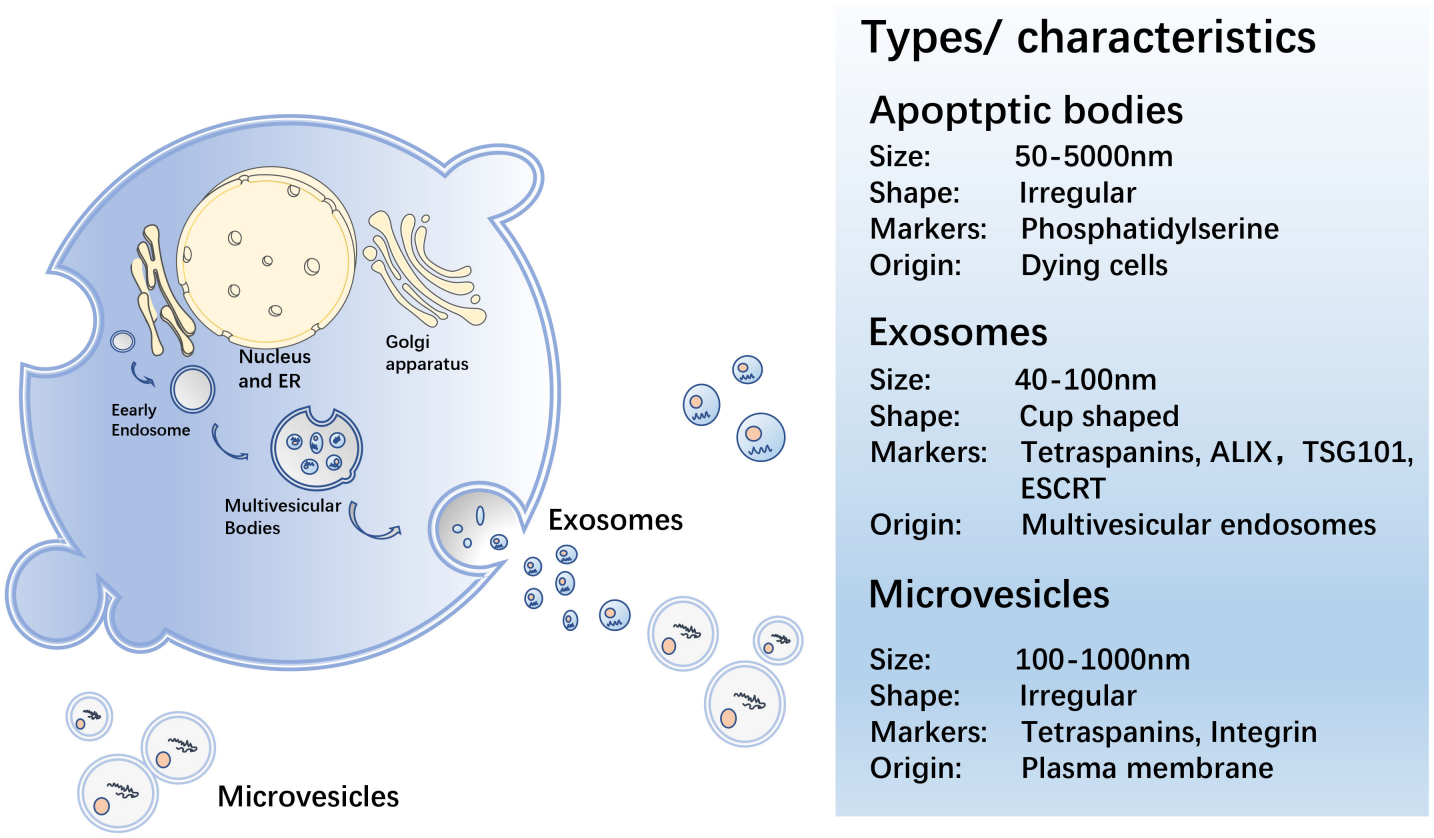
Biogenesis of EV.

EVs are secreted by all cells during normal, pathological, and physiological processes, and cells can transmit information *via* EVs in the form of lipids, proteins, or nucleic acids to achieve intercellular communication (22). EVs are spherical in shape, separated by a phospholipid bilayer that protect their load from enzymatic destruction during transfer from the donor to receiving cell. MVs, ApoBDs, and exosomes are the three primary EV groups (23) (**Figure 1**).

MV is a heterogeneous cell-derived membrane vesicle that blisters and extrudes straight outward from the cell surface before being released into the extracellular environment during a highly controlled process. Vesicle contents are determined by the type of cell they arise from (24). MVs transport membrane-derived receptors, cytokines, chemokines, cell signalling proteins, lipids, carbohydrates, and genetic material, including DNA and various types of RNA, such as mRNA and miRNA (25). When cells are stimulated by cell injury, pro-inflammatory stimuli, hypoxia, or oxidative and shear stress, MV shedding increases (26). MVs hold carriers that can be discharged and influence the extracellular environment by being ejected from the cell, or act by fusing with the target cells (24).

An ApoBD is a tiny vesicle linked to the cell membrane and discharged as a cellular vesicle after apoptosis. ApoBDs range in size from 50–5000 nm (27). Before ApoBD was revealed to be capable of delivering helpful molecules to healthy recipient cells, these vesicles were thought to be dead cell garbage bags (28). Exogenous apoptosis induction can be employed to kill cancer cells in the biomedical area. Repetitive blistering and contraction of apoptotic cells result in the creation of ApoBDs that are then recognized, phagocytosed, and eventually degraded by lysosomes (28). ApoBDs have a prolonged procoagulant impact on the cancer cells (29). Rapid clearance of apoptotic cells is critical in autoimmune diseases for developing immunological tolerance and avoiding inflammatory reactions, and clearance of ApoBD abnormalities may contribute to the development of autoimmunity (30, 31). Studies show that ApoBDs may play a major role in anti-cancer immunity; however, their method of action requires further investigation (32, 33).

Exosomes are 40–100 nm in diameter, generated by continuous invagination of the plasma membrane that merges with the cell membrane, and are discharged outside the cell by the process of cytosolic vomiting (34, 35). Exosomes, like other EV subtypes, can be found in the bodily fluids, such as blood, urine, saliva, and breast milk (36–39). Exosomes contain proteins, DNA, mRNA, miRNA, and lipids, and their molecular composition is derived from the source cells. Their characteristics can reflect the multiple physiological functions or pathological states of the progenitor cells, and their function is determined by the cells from which they originate (40). Exosomes play a vital role in intercellular communication, as well as in normal physiological responses and pathobiological processes. Annexins, flotillins, and tetraspanins (CD9, CD63, CD81, and CD82) (41) are the proteins that can be involved in intracellular assembly and transport. The tetraspanins, CD9, CD63, CD37, CD81, or CD82, are usually found in the membranes of exosomes and are, therefore, used as biomarkers for exosome identification (42). Exosomes have been reported to be involved in many biological processes, including the presentation of antigens in immune responses, angiogenesis promotion, and removal of undesirable proteins and RNA. They also play a role in various pathological processes, including tumour formation and metastasis (43–45). They, for example, are crucial for the transport of bioactive chemicals from the main tumour site to other cells and organs in the local and distant microenvironment (17). Current studies focus on exosomes, which contain multitudinous components, including miRNA, lncRNA, circRNA, proteins etc., as described in this review.

### 2.2 Isolation methods of EV

There are several methods available for the isolation of EVs, each with different advantages, disadvantages, and variations in the purity of EVs obtained (**Table 1**). The subtypes of EVs are characterized by their biogenesis, size, physical features, contents, and function (54). Manipulations are made based on their diameter, biochemical features, and surface indicators to better isolate exosomes from other components for more in-depth investigations (55). There are several approaches for separating exosomes, including differential and density gradient centrifugation and ultrafiltration, among others, each with their own separation principles, advantages, and disadvantages.

**Table 1:** Isolation method of EV.

EV Isolation methods	principle	Advantages	Disadvantages	Reference
Ultracentrifugation	Differential centrifugation using different centrifugal forces	Simple operation, no complex sample pre-treatment required	Exosome damage, time-consuming, non-exosomal impurities, equipment	(40, 46)
Density gradient	Buoyant density	High purification and enrichment of exosomes	Complexity, low recovery	(47)
Ultrafiltration	Difference in size	Many samples can be processed simultaneously	Sample loss, vesicle deformation	(48, 49)
Size-exclusion chromatography	Columns with polymer filled with anisotropic porosity	Economically beneficial	Complexity, specialized equipment, cost	(50)
Capture-based techniques	Binding to target proteins on the membrane surface	High purity, specificity	Cost, complexity	(51)
Microfluidics−based techniques	Manipulation of fluids in micro- and nano-scale space	Cost, efficient	Equipment, complexity	(52, 53)

Differential centrifugation is one approach used to separate exosomes in cell culture, wherein big particles and cell debris are isolated from the medium by centrifugation at 200–100,000 × *g* and exosomes recovered from the supernatant by centrifugation at 100,000 × *g* (46). This approach is most commonly employed; however, it is inefficient for separating exosomes, as it contains a combination of other components in the filtered product, is time-consuming, and requires specialized equipment (40).To overcome the limitations of differential centrifugation, samples can be separated using density gradient centrifugation. It has been demonstrated that density gradient centrifugation can separate subcellular components and improve particle separation performance based on buoyancy and density (47). This can improve yield, resulting in highly purified and concentrated exosomes.

Separation can also be performed based on EV size disparities. Ultrafiltration, which has a molecular weight cut-off of 10–100 kDa, concentrates exosomes from a large amount of raw material into a small sample volume for subsequent purification and is often conducted as the first step in the separation process (48). Filtration begins by removing cells and detritus from the sample, then concentration for free proteins and, ultimately, depending on the diameter of the exosome, filtration to retrieve the target exosome (49). Size-exclusion chromatography has the advantage over other techniques as it is cost-effective and non-destructive for the separation of the sample. Studies have shown that ultrafiltration technology is more efficient than centrifugation, allowing more particles to be separated in less time and improving the purification rate of exosomes (50).

Magnetic beads in capture-based techniques play a central role by binding to target proteins on the membrane surface. This technique is closely related to immunoaffinity and is used for the production of high-purity exosomes. Its advantage over other separation techniques is the ability to isolate specific exosomes having high purity using specific immune interactions between antibodies and antigens (51).

Because of their capacity to separate continuously at high rates, microfluidics-based procedures have distinct advantages, such as cheaper costs and shorter operating times. However, this approach has drawbacks of high equipment needs and operating complexity. Microfluidic techniques are currently fully integrated with size-based, immunoaffinity-based, and dynamic separations (52, 53, 56).

The optimum approach for exosome isolation should have the following characteristics: ease of use, efficiency, speed, and cost-effectiveness. The isolation method should not be harmful to exosomes. The advantages and disadvantages of each approach for isolating exosomes should analysed, and the limitations should be addressed further. Despite the fact that there are several separation methods, ultracentrifugation is still regarded as the gold standard and most prominent EV separation method, and many research extensively use this approach.

EVs are abundant in organisms and engage in a range of life activities, containing a diversity of proteins and genetic materials. They play a range of roles depending on their contents. Exosomes, for example, are crucial for the transport of bioactive chemicals from the main tumour site to other cells and organs in the local and distant microenvironment (17). Is it feasible to identify the contents of EVs in order to acquire more information about the tumour and better understand the response to immunotherapy with so many live things in EV?

## 3 EV as a biomarker in NSCLC immunotherapy

Exosomes with size more than 40 nm and less than 100 nm in diameter are still the focus of studies on EV as a biomarker. The function of exosomes as carriers of natural biomarkers in illness detection has attracted much attention. Exosomes are still being used in NSCLC immunotherapy to provide new information on future prognostic techniques.

Neoadjuvant therapy is a systemic anti-tumour medication administered to patients before surgery. Neoadjuvant therapy may involve chemotherapy, immunotherapy, targeted therapy, radiation, and other treatments depending on the kind of tumour involved. Exosomal miRNA-21, miRNA-222, and miRNA-155 have been shown to be useful biomarkers for the diagnosis and prognosis of patients with breast cancer receiving neoadjuvant chemotherapy (57). This shows that exosomes might play a key role during tumour neoadjuvant therapy; however, there is no relevant research in NSCLC.

### 3.1 Application of EV as biomarker for immunotherapy

PD-L1, which interacts with PD-1 to enhance tumour cell evasion and T cell inactivation, is an immunosuppressive chemical that tumour-derived EVs (TDEs) may carry and use as a modality for immunotherapy (58). For example, NKG2D ligands can aid tumour cells in evading immune surveillance when it is expressed on TDEs. NKG2D is a key recognition receptor for the identification and eradication of tumour cells (59). FASL expressed on TDEs can also encourage immune cells to die, which enhances the growth of tumours (60). Through these immunosuppressive chemicals, TDEs can act as indicators of tumour cells.

### 3.2 Application of EV as biomarker in tumor microenvironment

The tumour microenvironment (TME), which includes cancer-associated fibroblasts, adipocytes, neuroendocrine cells, and vascular and lymphatic networks, is the internal environment involved in the development and progression of tumours (61). According to previous studies, exosomes have a particularly significant influence on tumorigenesis, signalling, and progression. The diversity of tumorigenesis and tumour genetics is reflected in TDEs, which is mostly generated from the cell membrane and endosome of primitive tumour cells and include a wide variety of tumour antigens. TDEs show enormous promise as a biomarker for early cancer detection, diagnosis, and prognosis (62). To create a TME that promotes tumour cell survival and metastasis, tumour-derived exosomes are loaded with chemicals that inhibit immune responses and inflammation (63). Immune system responses to tumours can be boosted by exosomes produced in immune cells (64). Exosomes have been revealed to be crucial for coordinating intercellular communication as well as facilitating communication between cancer cells and the cells in the TME (65). As a result, tumour-derived exosomes are significant and have the potential to as new, minimally invasive biomarkers for cancer immunotherapy and are capable of taking part in the immunological TME (66).

TDEs participate in the TME, which allows them to play a dual function in the anti-tumour immunological mechanism. TDEs play a function in reducing immunological activity by stimulating the differentiation of immune-suppressive cells. In contrast, TDEs can also produce an inflammatory milieu that promotes continued tumour growth. It has been demonstrated that HCC-derived exosome high mobility group box 1 (HMGB1) causes the growth of immune cells that release IL-10 and impair CD8+ T cell activity (67). TDEs carry tumour-associated antigens on their surface that can trigger an immune response in the early stages of malignancies (68). They can play an important role in tumour immunotherapy by acting as effectors of immune cell activation, and B cells and dendritic cells and can induce an immune response by presenting antigens to activate T cells (69, 70). For instance, TDEs containing HSP70 can cause natural killer cells to operate as immune cells (71). Studies have shown that microenvironmental acidity and the release of EVs are related, and that microenvironmental acidosis of patients with cancer can cause an enhanced release of EVs. In order to diagnose and predict the development of cancer, EVs in the TME can be employed (72, 73). In conclusion, EVs and the TME are strongly linked to the onset and management of cancer; however, more research is still needed to fully understand the dual function of EVs in the TME.

We reviewed the registered clinical trials (clinicaltrials.gov) on the use of EVs or exosomes in NSCLC (**Table 2**). We found some patterns where clinical studies on EVs in NSCLC focused on its role as a biomarker in early diagnosis and in the therapeutic efficacy of drugs during the mid-to-late stages. In addition, these studies have mainly focused on the last three years, and the materials examined were primarily blood and alveolar lavage fluid. The mRNA, lncRNA, proteins, and other compounds are among those that were investigated. Development in this area of research is summarized in section 4.

**Table 2:** Clinical trials registered on clinicaltrials.gov on EV or exosome for NSCLC.

No.	Trial name	NCT#	Location	Estimated or Actual Enrollment	First Posted date	Source of EV	Detect contents of EV
1	Comparision of Various Biomarkers Between Peripheral and Pulmonary Blood	NCT05587114	Korea University Guro Hospital	150 patients after lung cancer surgery	19-Oct-22	Blood plasma	various biomarkers
2	Neoadjuvant Lazertinib Therapy in EGFR-Mutation Positive Lung Adenocarcinoma Detected by BALF Liquid Biopsy	NCT05469022	Konkuk University Medical Center	40 NSCLC patient with EGFR gene mutations	21-Jul-22	bronchoalveolar lavage fluid	EGFR genotyping
3	Extracellular Vesicles and Particles (EVP) as Biomarkers of Recurrence in Non-Small Cell Lung Cancer	NCT05424029	Memorial Sloan Kettering Cancer Center	200 with NSCLC patients after surgery	21-Jun-22	bronchial washings	vesicles number
4	Exosomes Detection for the Prediction of the Efficacy and Adverse Reactions of Anlotinib in Patients With Advanced NSCLC	NCT05218759	Shanghai Chest Hospital	30 patients with advanced NSCLC and treatment with Anlotinib	1-Feb-22	Blood plasma	microRNA
5	Molecular Profiling of Exosomes in Tumor-draining Vein of Early-staged Lung Cancer (ExOnSite-Pro)	NCT04939324	CHU de Limoges	30 with NSCLC patients after surgery	25-Jun-21	Blood and tumor tissue	Size distribution, molecular profiling of exosome
6	Improving the Early Detection of Lung Cancer by Combining Exosomal Analysis of Hypoxia With Standard of Care Imaging	NCT04629079	King’s College London	800 patients with lung cancer	16-Nov-20	Blood plasma	pre-microRNA
7	An Observational Study to Evaluate the Clinical Utility of the Oncomine Precision Assay Within the Exactis Network	NCT04564079	Centre hospitalier universitaire Dr-Georges-L.-Dumont	200 patients with stage IIIb/IV NSCLC	25-Sep-20	Blood plasma	sensitivity and specificity of mutation
8	Multicenter Clinical Research for Early Diagnosis of Lung Cancer Using Blood Plasma Derived Exosome	NCT04529915	Korea University Guro Hospital	normal people (n = 150) and lung cancer patients (n = 320)	28-Aug-20	Blood plasma	Protein
9	The Study of Exosome EML4-ALK Fusion in NSCLC Clinical Diagnosis and Dynamic Monitoring	NCT04499794	Cancer Institute and Hospital, Chinese Academy of Medical Sciences	75 patients with stage IIIB-IV unresectable NSCLC	5-Aug-20	Blood plasma	EML4-ALK fusion
10	Prediction of Immunotherapeutic Effect of Advanced Non-small Cell Lung Cancer	NCT04427475	Fudan University	200 patients with advanced NSCLC under immunotherapeutic	11-Jun-20	Blood plasma	microRNA
11	Validation of Multiparametric Models and Circulating and Imaging Biomarkers to Improve Lung Cancer EARLY Detection	NCT04323579	Istituto Clinico Humanitas	150 lung cancer patients and 120 matched controls	26-Mar-20	Blood plasma	antigens
12	Circulating and Imaging Biomarkers to Improve Lung Cancer Management and Early Detection	NCT04315753	Istituto Clinico Humanitas	2000 old and smoking patients with lung cancer	20-Mar-20	Blood plasma	antigens
13	Clinical Study of ctDNA and Exosome Combined Detection to Identify Benign and Malignant Pulmonary Nodules	NCT04182893	Shanghai Chest Hospital	400 people with 0.5-3cm pulmonary lesions in chest CT	2-Dec-19	Blood plasma	mRNA
14	Serum Exosomal Long Noncoding RNAs as Potential Biomarkers for Lung Cancer Diagnosis	NCT03830619	Wuhan Union Hospital, China	1000 patients with lung cancer	5-Feb-19	Blood plasma and cell culture media	lncRNA
15	Combined Diagnosis of CT and Exosome in Early Lung Cancer	NCT03542253	Second Affiliated Hospital of Soochow University	80 patients with Chest or LDCT examination for the first time found 5-30mm pulmonary nodule	31-May-18	cancer and paracancerous tissue	microRNA
16	Detection of Either the EML4-ALK Gene Rearrangements or the T790M EGFR Mutation in the Plasma of Advanced NSCLC Patients	NCT03236675	Exosome Diagnostics, Inc.	60 patient with IIIB-IV NSCLC	2-Aug-17	Blood plasma	EGFR genotyping
17	Olmutinib Trial in T790M (+) NSCLC Patients Detected by Liquid Biopsy Using BALF Extracellular Vesicular DNA	NCT03228277	Konkuk University Medical Center	25 patients with T790M-positive NSCLC	24-Jul-17	bronchoalveolar lavage fluid	DNA
18	Clinical Research for the Consistency Analysis of PD-L1 in Lung Cancer Tissue and Plasma Exosome Before and After Radiotherapy	NCT02869685	Xinqiao Hospital of Chongqing	200 patients with NSCLC before and after radiotherapy	17-Aug-16	Blood plasma	mRNA

## 4 Different components of EV as biomarker in NSCLC immunotherapy research

**Figure 2:**
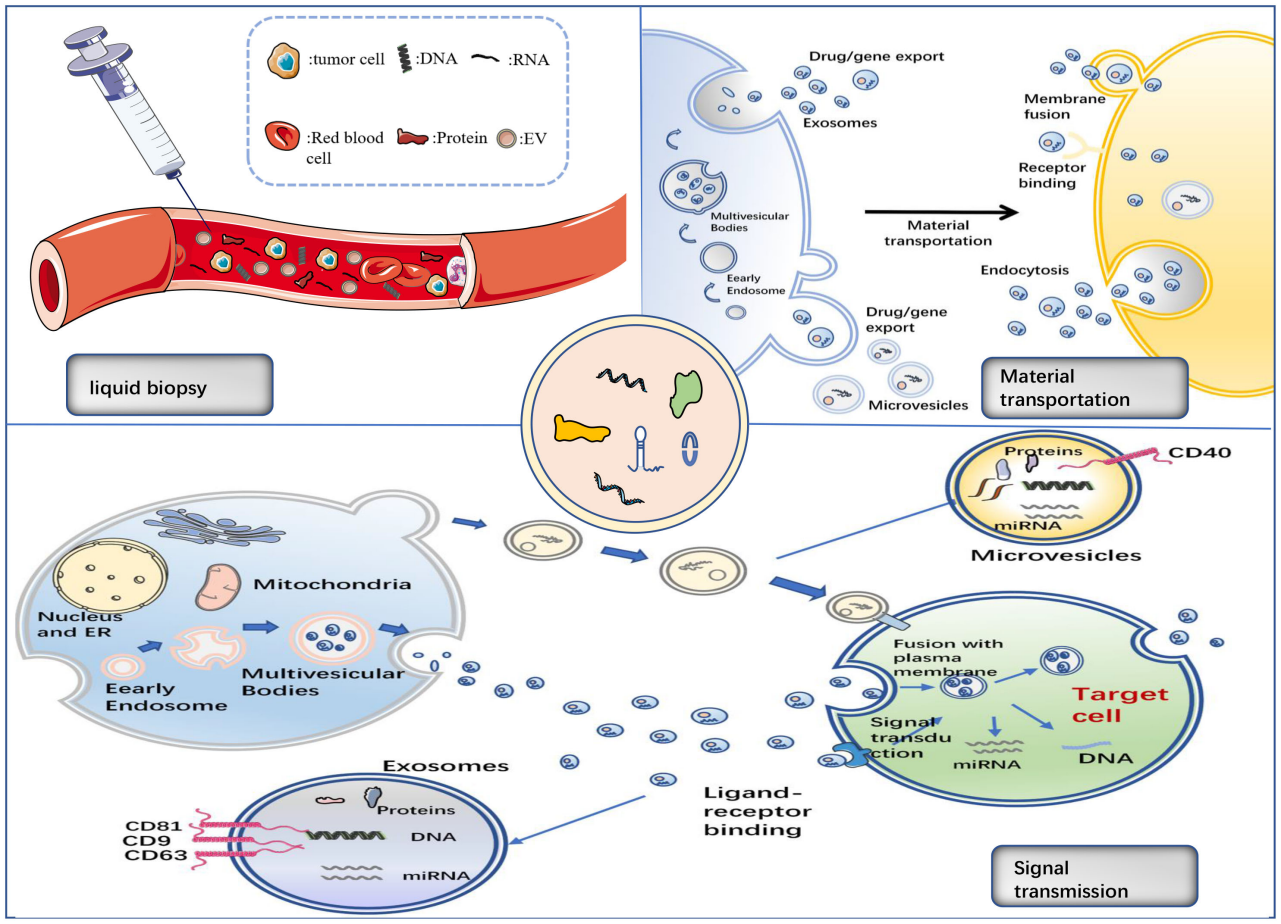
The functions of EV.

As previously stated, an EV comprises DNA, RNA, proteins, lipids, and other components. These elements are involved in tumorigenesis and can have a crucial role in immunotherapy during the early diagnosis, treatment, and prognosis of cancer. The primary focus of biomarker research in immunotherapy is on miRNA, lncRNA, and proteins, which we will discuss in this section separately (**Table 3**, **Figure 2**).

**Table 3:** Different components of EV as biomarker in NSCLC immunotherapy research.

Biomarkers	Expression	Recipient cell	Target	Function	Reference
miR-21	Up	HBE cells	STAT3	Blocking angiogenesis	(74)
miR-494 miR-524-3p	Up	AD cells	LnStr, LFb	Regulating organs prior to tumour metastasis	(75)
LINC00301	Up	NSCLC cells	TGF-β	Impact on NSCLC development	(76)
lncRNA UFC1	Up	NSCLC cells	EZH2	Impact on NSCLC development	(77)
CircNDUFB2	Up	NSCLC cells	IGF2BPs	Stimulates anti-tumour immunity	(78)
has-circRNA-002178	Up	AD cells	/	Promoting PDL1/PD1 expression in lung adenocarcinoma	(79)
circUSP7	Up	NSCLC cells	CD8+ T cells	Promotion of immunosuppression	(80)
PKM2	Up	NSCLC cells	/	Promotes NSCLC cell proliferation and cisplatin resistance	(21)
PLA2G10	Up	NSCLC cells	/	Negative correlation with NSCLC prognosis	(81)
GCC2	Up	NSCLC cells	/	Early diagnostic biomarkers for NSCLC	(82)
GCC2-ALK	Up	NSCLC cells	ALK	For NSCLC diagnosis and treatment	(83)
LBP	Up	NSCLC cells	/	Diagnosis of metastatic NSCLC	(84)

### 4.1 RNA

The miRNAs can influence further tumour progression in immunotherapy of lung cancer by modulating lung cancer immune checkpoints and acting as a biomarker. It was found that miRNA-200 levels were closely associated with PD-L1 expression (85). Four miRNA signatures in serum (miR-193b, miR-301, miR-141, and miR-200b) can be used to differentiate NSCLC from non-cancerous individuals (86). Exosomal miRNAs are critically associated with the development, spread, and metastasis of NSCLC. Previous studies have shown that exosomal miRNAs can promote angiogenesis. For example, STAT3-regulated exosomal miR-21 promotes angiogenesis and induces malignant transformation of human bronchial epithelial cells (74). In addition, exosomal miRNAs are involved in the process of tumour metastasis. The AD cell-derived exosomes, miR-494 and miR-524-3p, have been reported to regulate pre-metastatic organoids (75).

T cells are immunosuppressed by myeloid-derived suppressor cells in malignancies. According to previous studies, lncRNAs control the ability of myeloid-derived suppressor cells to inhibit the immune system in the TME, which influences the development of lung cancer. In other studies, patients with lung cancer were found to have lower expression levels of *HOTAIRM1* in their peripheral blood cells, and when this gene was overexpressed, the immunosuppressive properties of myeloid-derived suppressor cells decreased and tumour immune responses strengthened (87). Additionally, lncRNAs affect tumour development by controlling T cell activity. According to previous reports, NSCLC has a high expression of *LINC00301*. By concentrating on TGF-β, linC00301 can decrease the levels of CD8+ T cells, accelerating the development of NSCLC (76). Exosomal lncRNAs are directly related to the development of NSCLC. Zang et al. (77) discovered that lncRNA UFC1 expression levels were raised in the tumour tissues, serum, and serum exosomes of patients with NSCLC. High UFC1 levels were linked to tumour invasion. Researchers discovered that exosomal-delivered UFC1 might bind to EZH2, downregulate *PTEN* gene expression, and activate the PI3K/Akt signalling pathway, encouraging tumorigenesis in NSCLC.

In the treatment of NSCLC using immunotherapy, circRNA is also crucial. It was discovered that circNDUFB2 had a poor correlation with NSCLC’s malignant characteristics. By controlling the cellular immune response, circNDUFB2 was able to stimulate anti-tumour immunity throughout the development of NSCLC (78). According to Wang et al., the expression of has-circRNA-002178 was noticeably elevated in AD tumour tissues. T cell failure might result from the delivery of has-circRNA-002178 to CD8+ T cells, which would cause PD-1 expression to be induced *via* exosomes (79). Exosomal circRNA have also been implicated in the advancement of NSCLC in several investigations. The release of IFN-γ, TNF-α, granzyme B, and perforin by CD8+ T cells has been shown to be suppressed by the tumour-derived exosome, circUSP7, preventing CD8+ T cells from performing their normal role. As a result, circUSP7 may encourage patients with NSCLC to get anti-PD-1 treatment (80).

### 4.2 Protein

A recent study revealed a unique mechanism for the propagation of drug resistance in solid tumours (21): Hypoxia-induced exosomes transfer PKM2 to susceptible NSCLC cells and transmit cisplatin resistance. Exosomal PKM2 might be a viable biomarker and therapeutic target for NSCLC cisplatin resistance. Exosomal PLA2G10 protein levels were considerably greater in NSCLC samples than in healthy samples (81). Furthermore, patients with NSCLC with greater amounts of exosomal PLA2G10 protein had poorer overall and relapse-free survival rates. These findings show that the PLA2G10 protein found in exosomes might be a helpful biomarker for identifying and predicting survival in individuals with NSCLC. Exosomal GCC2 considerably changes with pathological stage, has high specificity and sensitivity in detecting early-stage NSCLC, and is likely to contribute greatly to the diagnosis of asymptomatic patients with early-stage lung cancer in routine screening (82). Interestingly, GCC2-ALK fusion proteins were discovered in individuals with NSCLC in a prior study (83). GCC2-ALK overexpression has been demonstrated to trigger downstream ALK signalling, which can be blocked by ALK inhibitors, such as crizotinib and ceritinib. These findings show that GCC2 is a viable target for NSCLC diagnosis and/or therapy. Wang et al. (84) used proteomic techniques to look for diagnostic markers for metastatic NSCLC and discovered significant differences in the levels of LBP in exosomes and circulation of patients with metastatic and non-metastatic NSCLC, indicating that LBP has the potential to act as a metastatic NSCLC biomarker.

### 4.3 Multi-omics approach for EV

As cancer tissue samples are very variable and incredibly complicated, different technological techniques and data formats might produce disparate outcomes. As a result, multi-omics analysis-employing approaches, from genomics, transcriptomics, epigenomics, proteomics, metabolomics, and other areas of histology, are seen as critical to the advancement of precision treatment in cancer.

Luo et al. (88) integrated the analysis of transcriptome and proteome data to reveal the diverse functions of exosomal-enriched RNAs and proteins, many of which are associated with tumorigenesis. Importantly, several human lung AD stem-like cell markers identified were highly expressed in AD stem-like cell-derived exosomes and associated with poor survival, which may thus serve as promising liquid biopsy biomarkers for lung AD diagnosis. Through using weighted gene co-expression network analysis, Chen et al. (89) identified a series of known, conserved, and novel exosomal miRNAs associated with the severity of anxiety and depression, as well as concentrations of the neurotransmitters, GABA, choline, and serotonin. Soon, Sun Kim et al. (90) identified differentially expressed lncRNAs in HCC and healthy donor EVs, and selected *LINC00853* as a novel biomarker for early detection of HCC.

The advancement of multi-omics technologies has not only increased our understanding of tumour biology but also uncovered intriguing new biomarkers and therapeutic targets. However, no research has focused on immunotherapy in NSCLC. As a result, more research using multi-omics approaches in NSCLC is needed, and the integration of multi-omics data for NSCLC discrimination could become a growing trend.

### 4.4 Other roles of EV

Exosome indicators are also important in liquid biopsies. Circulating tumour cells, circulating tumour DNA, circulating cell-free DNA, miRNA, and non-coding RNA are all used in liquid biopsies to help diagnose and treat cancer (63). Flow-based traditional tissue biopsies are invasive, can result in complications that include bleeding and infection, and may be less accurate at forecasting treatment results than liquid biopsies (16). Additionally, it might not accurately anticipate the outcome of therapy. Conversely, non-invasive liquid biopsies using urine-derived exosomes in patients with NSCLC can identify exosomes from bodily fluids and predict therapy responses (63).

Few studies currently use EVs as ICI biomarkers for liquid biopsies during exosomal immunotherapy. More research has been done on EVs that contain PD-L1. According to one study, releasing the gene for PD-L1-containing exosomes in a mouse model 1 reduced tumour development (91). According to another study, the forced expression of PD-L1 on cells devoid of PD-L1 and administration of PD-L1-containing exosomes to NSCLC tissue both prevented the formation of tumour tissue (92). These data imply that exosomal PD-L1 should be investigated further as a biomarker for NSCLC therapy.

Different subpopulations of exosomes are loaded with various miRNAs, which may have various biological activities. EVs also include a range of bioactive compounds. The gold standard for the clinical detection of cancer using liquid biopsy is circulating DNA, which is secreted by cells along with EVs and used to diagnose NSCLC (93, 94). Through the use of EVs, tumour cells can also avoid immune monitoring, facilitating distant metastasis. Cancer-causing proteins are carried by tumour cell EVs and cause healthy fibroblasts to develop carcinogenesis (95, 96). Additionally, tumour cell EVs contribute to targeted cancer therapy. Tumour cell EVs have reportedly evolved into one of the techniques used for evaluating the efficacy of targeted therapy. For instance, the low sensitivity in circulating DNA liquid biopsies has been enhanced by the introduction of exosomal RNA as a biomarker to identify T790M mutations in the *EGFR* gene (97, 98). EVs can contribute to medication delivery because they can load biomolecules with parental cell selectivity. The particular binding of EVs to cell surface receptors for message transmission, EV signalling, and substance transport by membrane fusion and endocytosis are some of the methods used to transport EV contents (99, 100). According to previous studies, using EVs to transport macromolecular medicines is more reliable and has a more powerful impact on anticancer treatment (101). Acidification of the TME also enhances EV fusion with tumour cells by encouraging EV absorption. This underlines the inherent ability of EVs to transport macromolecules (102, 103). It has been noted that transfection is a successful technique for introducing RNA medicines into EVs. The viability of gene delivery by EVs was shown when siRNAs targeting *RAD51* and *RAD52* were transfected into EVs and triggered gene silencing *via* transport to target cells (104). The viability of gene immunotherapy is increased by EV-mediated gene delivery. Similar to this, EVs were shown to enhance the endocytosis and stability of protein drug delivery during cancer treatment (105). EVs offer distinct benefits and promise in the detection and treatment of cancer, and more research is still needed to determine how well they serve as delivery vehicles.

## 5 Conclusion

Immunotherapy has emerged as one of the most significant medical treatment methods for NSCLC, and novel immunotherapeutic approaches are being developed. EVs are gaining popularity as a component of liquid biopsies. EVs have been demonstrated to play an important role in various immunotherapeutic marker investigations, offering a novel viewpoint on the diagnosis and prognosis of NSCLC. We show how EVs play an important role in the prevention, diagnosis, and treatment of NSCLC by secreting miRNAs, lncRNAs, circRNAs, and proteins. More studies about EVs are being conducted and we believe that they will play an important role as a biomarker for NSCLC immunotherapy and improve patient prognosis.

## Author contributions

YG, TY and SF wrote the paper. BL and JW reviewed and edited the manuscript. XJ, HS and GW prepared the figure and table. All authors contributed to the article and approved the submitted version.
